# Esthetic Concerns and Acceptability of Treatment Modalities in Primary Teeth: A Comparison between Children and Their Parents

**DOI:** 10.1155/2016/3163904

**Published:** 2016-06-29

**Authors:** Sharat Chandra Pani, Abdulrahman Al Saffan, Sultan AlHobail, Fares Bin Salem, AlBara AlFuraih, Mohammad AlTamimi

**Affiliations:** ^1^Department of Pediatric and Preventive Dentistry, Riyadh Colleges of Dentistry and Pharmacy, Riyadh 11681, Saudi Arabia; ^2^Department of Preventive Dentistry, Riyadh Colleges of Dentistry and Pharmacy, Riyadh 11681, Saudi Arabia; ^3^Riyadh Colleges of Dentistry and Pharmacy, Riyadh 11681, Saudi Arabia

## Abstract

*Background and Aim*. Esthetic concerns in primary teeth have been studied mainly from the point of view of parents. The aim of this study was to study compare the opinions of children aged 5–8 years to have an opinion regarding the changes in appearance of their teeth due to dental caries and the materials used to restore those teeth.* Methodology*. A total of 107 children and both of their parents (*n* = 321), who were seeking dental treatment, were included in this study. A tool comprising a questionnaire and pictures of carious lesions and their treatment arranged in the form of a presentation was validated and tested on 20 children and their parents. The validated tool was then tested on all participants.* Results.* Children had acceptable validity statistics for the tool suggesting that they were able to make informed decisions regarding esthetic restorations. There was no difference between the responses of the children and their parents on most points. Zirconia crowns appeared to be the most acceptable full coverage restoration for primary anterior teeth among both children and their parents.* Conclusion.* Within the limitations of the study it can be concluded that children in their sixth year of life are capable of appreciating the esthetics of the restorations for their anterior teeth.

## 1. Introduction

Esthetic dentistry is today an essential component of modern dental practice. However while there is a considerable amount of literature available on the subjective influences in adults little is known about esthetics in children [1, 2].

Early childhood caries (ECC) is defined as the presence of one carious lesion or more on the teeth of children younger than 71 months of age [3]. The labial surface of the upper anterior teeth is one of the most commonly affected surfaces in ECC, which results in the visibility of these carious lesions [1, 4]. There have been several esthetic treatment modalities which have been used to treat carious lesions in the primary teeth in very young children. There have been studies that have looked at parental satisfaction with these restorations and few studies have attempted to study the differences between parents and dentists on esthetic treatment [4–7].

The knowledge of the children's aesthetic perception is relevant to pediatric dentists because children are conscious about their dental aesthetic appearance and that of the other children [8, 9]. While the traditional concept of Jean Piaget stated that a child's perception of self and care about their appearance only developed by the age of 8 years; there have been recent studies in the field of child psychology that have challenged this concept, showing that, with increased media exposure, children as young as 3–5 years of age have a sense of consciousness of body image [9–11]. These studies have focused on the self-perception of children below the age of five years as compared to that of their parents from the point of view of obesity and body image [10, 11]. However, almost no attempt has been made to study the perception of children or any differences they may have with their parents on the matter of their dental esthetics [1].

The aim of this study was to study the ability of children in their sixth year of life to have an opinion regarding the changes in appearance of their teeth due to dental caries and the materials used to restore those teeth. The study also aimed to compare the opinion of the children with that of their parents.

## 2. Methodology

A total of 107 children and both of their parents (*n* = 321), who were seeking dental treatment for badly destructed teeth, were included in this study. The aim of the study was explained to the parents and informed consent forms for the parents and the children (signed by the parents) were obtained before proceeding with the study. The study received ethical approval from the research center of the Riyadh Colleges of Dentistry and Pharmacy (USRP/13/131).

### 2.1. Sample Selection

All the children and their parents who met the inclusion criteria and were seeking subsidized treatment were screened through the community outreach program of the King Salman Center for Children Health. In order to achieve a good sample size, all parents and children screened between March 2014 and September 2015 were included in the study.

### 2.2. Sample Power Calculation

A post hoc sample power calculation was done using the G-Power sample power calculator (University of Kiel, Kiel, Germany). The power of the sample with an effect size of 0.8 was determined to be 0.957 with an alpha of 0.05.

### 2.3. Preparation of the Instrument

A questionnaire in Arabic was prepared which recorded the demographic characteristics and the past dental experience of the family. The pictures of carious lesions and their treatment were selected from the archives of the Department of Pediatric Dentistry and edited using Adobe Photoshop CS5 picture editing software (Adobe Corp., San Jose, CA, USA). The final pictures were arranged in the form of a presentation on Microsoft office Powerpoint*™* (Microsoft Corp., Redmond, WA, USA) and shown to each parent and the child. The purpose of each section of the presentation was explained to the parent and the child by a single examiner (SH) to rule out examiner bias.

**Figure 1:**
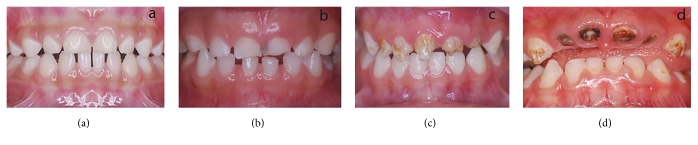
Point at which the respondent would visit the dentist. (a) No caries, (b) initial cavitation of the tooth, (c) deep caries with partial destruction of the crown, and (d) complete destruction of the anterior teeth.

The presentation comprised three parts; the first part was designed to evaluate at what point the parent and/or the child would visit the dentist for the treatment of caries in the anterior teeth, while the other two parts attempted to study the acceptability of different treatment modalities. The first part showed the parent or the child a series of pictures of the anterior teeth, ranging from complete crown destruction to normal teeth (Figure 1) and the respondent was asked to choose what level of caries was acceptable to them before they would seek treatment. The pictures were shown separately to both parents and the child, while the demographic part of the questionnaire was only administered to the parents.

**Figure 2:**
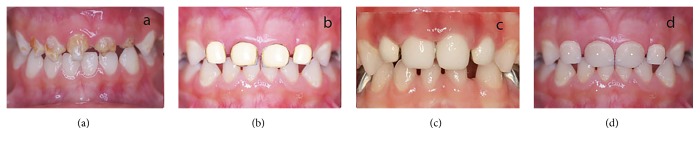
Type of treatment acceptable for deep anterior caries. (a) No treatment, (b) open (resin) faced stainless steel crown, (c) strip crown, and (d) zirconia crown.

The second part of the presentation comprised a series of four photographs that depicted badly destructed anterior teeth and the restoration of these teeth by open (resin) faced stainless steel crowns, composite “strip” crowns, and zirconia crowns. Pictures of the treatment were edited using Adobe Photoshop CS5 (Adobe Corp., San Hose, CA, USA) so as to ensure that the focus of the treatment remained on the anterior teeth (Figure 2). Parents and children were then shown the edited pictures and asked to rate the picture as esthetically acceptable (1) or unacceptable (0).

**Figure 3:**
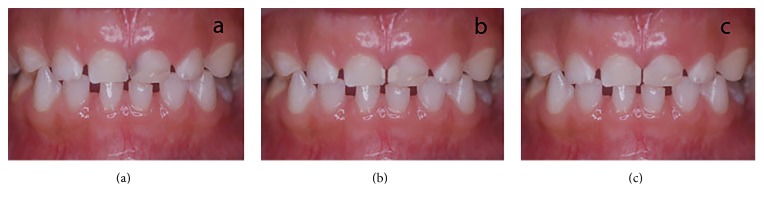
Type of treatment acceptable for anterior teeth with mild caries. (a) No treatment, (b) glass ionomer cement restoration, and (c) composite restoration.

The third part comprised a picture of anterior teeth with mild caries. Adobe Photoshop CS5 (Adobe Corp., USA) was used to superimpose restoration from other patients to images of the tooth restored with glass ionomer cement and composite (Figure 3). The parents and children were similarly asked to rate the picture as esthetically acceptable (1) or unacceptable (0).

### 2.4. Validation of the Tool

The presentation was repeated after 6 weeks to a group of 20 children and their parents to test for validity of the tool. The responses were compared between the first visit and recall visit and Cronbach's alpha statistic was applied. The presentation had an overall alpha value of 0.88 for fathers, 0.91 for mothers, and 0.71 for children suggesting that the tool had good reproducibility for the parents and acceptable reproducibility for the children. Cronbach's alpha of each of the components of the questionnaire is summarized in Table 1. The initial responses of the subjects used for validity assessment (*n* = 60) were included in the final analysis.

### 2.5. Administration of the Tool

The tool was tested on parents and children separately. The tool was tested on each parent individually while the other parent waited outside with the child. The child had the presence of both parents while answering but was not prompted by either parent.

### 2.6. Analysis of the Results

The data collected was encoded into Microsoft Excel and IBM-SPSS ver. 21 (IBM Corp., Armonk, NY) and statistical analyses were applied. The Kruskal-Wallis test was applied to test the significance of difference between the responses of the parents and the children and also to test the significance of difference between responses to each question. The level of significance for this test was set at *p* < 0.05. Multiple Mann-Whitney *U* tests with Bonferroni correction were used as post hoc tests to measure for intragroup variation. Based on the correction the level of significance for the post hoc test was set at *p* < 0.01.

## 3. Results

The children were in their sixth year of life with a mean age of 6.28 years (±SD .65). The parents of the children were aged between 21 years and 55 years, with mothers (mean age 32.8 ± 5.2) being younger than the fathers (mean age 39.74 ± 6.2).

When the dental experience of the population was examined 95% the parents and half of the children had visited the dentist previously (Table 2). A total of 104 fathers (97.2%) and 105 mothers (98.1%) stated that they were concerned about the esthetics of their child's teeth. A total of 100 fathers (93.5%) and 103 mothers (96.3%) stated that they wished to improve the esthetics of their children's teeth.

When the respondents were shown pictures of different carious lesions of the anterior teeth, most of the respondents stated that they would only visit the dentist when there was a frank cavitation of the tooth. Significantly more mothers stated that they would seek treatment for white spot lesions and initial cavitation of anterior teeth than either fathers or children (Table 3).

When the respondents were shown the different options available for the treatment of badly destructed anterior teeth zirconia crowns were the most esthetically accepted treatment for most of the respondents. There was no significant difference between parents and their children in the acceptability of any of the treatment modalities except for open faced crowns, which were significantly more acceptable to children than their parents (Table 4). No significant differences were observed between the other treatment modalities shown to the respondents (Table 4). When shown the treatment options for mild anterior caries children were significantly more likely than their parent to choose no treatment for the lesion. Composite resins were significantly more acceptable to parents than their children. However, the same was true for glass ionomer. Significantly more children were willing to accept no treatment than their parents (Table 5).

## 4. Discussion

The issue of esthetics in very young children is one that has received a lot of attention in psychology literature [1, 9]. The same, however, is not true for dental literature. Few studies have attempted to study esthetics from the point of view of the child, choosing instead to focus on the parents' perception of their children's esthetics [4]. In this regard this study is one of the first studies to attempt to understand the esthetic needs of children and how this applies to the restoration of destructed anterior teeth.

There are several options available for the restoration of badly decayed carious anterior teeth including open faced crowns, composite strip crowns, and composite resin and glass ionomer cement for less severely decayed teeth [12]. In our study we chose to provide children and parents who were seeking to treat their anterior teeth with a selection of these different treatment options and study variations between them if any. Interestingly we found that there was no difference in opinion between children and their parents on most treatment options. Differences were however seen regarding the treatment options for initial decay in anterior teeth where children were significantly more likely to accept no treatment than their parents.

Glass ionomer restorations are often recommended by pediatric dentists given their fluoride release and cariostatic properties [12]. Despite the apparent acceptability of the glass ionomer cement, composite resins were clearly the most preferred restoration among parents and their children. These results are in keeping with findings from pediatric literature that composite resins are the most acceptable esthetic restoration in young children [8, 13].

For the management of deep anterior caries, parents and children alike found the images of zirconia crowns to be the most acceptable restoration for badly destructed anterior teeth. This is interesting as there have been several recent articles suggesting that the zirconia crowns may be a strong and esthetically superior restoration for badly destructed anterior crowns [14, 15]. The low acceptability of open resin faced stainless steel crowns in this study are in contrast to those of a previous study [2]. This seems to suggest that, as more esthetic options become available, parents and children will have higher esthetic expectations for the treatment of anterior primary teeth.

Evaluating the stage at which the children and their parents would seek treatment, we found that parents were more concerned about initial caries lesions than children. This is in keeping with the findings of Schulman et al. who in a study on tooth color perception found that there could be a significant difference in how tooth color was perceived by the parent and the child [16].

The very young child is often neglected by the dentist while choosing a treatment plan. This is often justified by authors who quote the early work of Piaget to state that children below the age of eight are not concerned about their body image. However, recent work in psychology has shown that consciousness about self can begin as early as 3–5 years of age [9]. This has been attributed to the increasing role of media, television, and exposure to a concept of “ideal beauty” from a very young age [10, 17]. The findings of our study show that while children from a young age are able to appreciate and have an opinion about dental esthetics, their opinion often agrees with that of their parents.

The results of this study must be viewed in the light of certain limitations. The study focused only on parents seeking treatment for decayed anterior teeth. This decision was taken because, given the high caries rate in Saudi Arabia [18], it was felt that such a population would be more representative than a caries-free population. The study also focused on a limited set of treatment options which were felt to be the most commonly used in the study population. Given the fact that the choice of esthetic restoration was the primary focus of the study, neither parents nor children were given the option of extracting the tooth. The results of the study must be viewed keeping in mind the fact that options such as preveneered crowns and compomers were not included in the study. More importantly, the use of photographic modification software to modify the photographs of the restoration must be kept in mind while viewing the reliability of the results. It must also be remembered that the sample chosen was a convenience sample and may not be reflective of all children. Lastly it must be remembered that the validity statistics of the tool for the children, while acceptable, are not as good as that for their parents.

## 5. Conclusions

Within the limitations of the study it can be concluded that children in their sixth year of life are capable of appreciating the esthetics of the restorations for their anterior teeth.

## Figures and Tables

**Table 1:** Validity statistics of the tool^*∗*^.

	Child	Mother	Father
Point at which you would visit the dentist	0.69	0.88	0.87
Treatment of deep anterior caries	0.76	0.94	0.86
Treatment of mild anterior caries	0.68	0.87	0.91
Overall validity	0.71	0.91	0.88

^**∗**^Calculated using the Cronbach's alpha.

**Table 2:** Past dental experience of the population.

	Child	Mother	Father
Previously visited the dentist	55 (51.4%)	94 (87.9%)	95 (88.8%)
Orthodontic treatment	0 (0%)	22 (20.6%)	5 (4.7%)
Esthetic anterior restoration	8 (7.5%)	40 (37.4%)	32 (29.9%)
Esthetic posterior restoration	27 (25.2%)	71 (66.4%)	65 (60.7%)

**Table 3:** Point at which the population would visit the dentist.

	Child	Mother	Father
Complete destruction of crown	48 (44.9%)^a^	38 (35.5%)^a^	38 (35.5%)^a^
Deep caries with partial destruction of crown	31 (29.0%)^a^	22 (20.6%)^a^	34 (31.8%)^a^
Initial cavitation of tooth	21 (19.6%)^a^	40 (37.4%)^b^	22 (20.6%)^a^
No caries on the tooth	6 (5.6%)^a^	7 (6.5%)^a^	12 (11.2%)^b^
Invalid/no response	1 (0.9%)^NA^	0 (0%)^NA^	1 (0%)^NA^

Based on the Kruskal-Wallis test with Mann-Whitney *U* test for post hoc comparisons.

Difference in superscript indicates significant differences between children and their parents.

Significance calculated at *p* < 0.01 after applying Bonferroni correction for multiple nonparametric comparisons.

**Table 4:** Esthetic acceptability of treatment for anterior teeth with deep caries.

	Child	Mother	Father
No treatment	5 (4.7%)^a^	5 (4.7%)^a^	4 (3.7%)^a^
Open (resin) faced crown	11 (10.3%)^a^	5 (4.7%)^b^	4 (3.7%)^b^
Strip crown	19 (17.8%)^a^	20 (18.7%)^a^	16 (18.7%)^a^
Zirconia crown	75 (70.1%)^a^	87 (81.3%)^a^	85 (79.4%)^a^

Based on the Kruskal-Wallis test with Mann-Whitney *U* test for post hoc comparisons.

Difference in superscript indicates significant differences between children and their parents.

Significance calculated at *p* < 0.01 after applying Bonferroni correction for multiple nonparametric comparisons.

**Table 5:** Esthetic acceptability of treatment for anterior teeth with mild caries.

	Child	Mother	Father
No treatment	21 (19.6%)^a^	5 (4.7%)^b^	11 (10.3%)^ab^
Composite	87 (81.3%)^a^	100 (93.5%)^a^	100 (93.5%)^a^
Glass ionomer	47 (43.9%)^a^	55 (51.4%)^a^	61 (57.0%)^a^

Based on the Kruskal-Wallis test with Mann-Whitney *U* test for post hoc comparisons.

Difference in superscript indicates significant differences between children and their parents.

Significance calculated at *p* < 0.01 after applying Bonferroni correction for multiple nonparametric comparisons.
